# Tyrosine kinases in rheumatoid arthritis

**DOI:** 10.1186/1476-9255-8-21

**Published:** 2011-08-24

**Authors:** Hiroshi Okamoto, Akiko Kobayashi

**Affiliations:** 1 Minami-Otsuka Institute of Technology, Minami-Otsuka Clinic, Tokyo, Japan

## Abstract

Rheumatoid arthritis (RA) is an inflammatory, polyarticular joint disease. A number of cellular responses are involved in the pathogenesis of rheumatoid arthritis, including activation of inflammatory cells and cytokine expression. The cellular responses involved in each of these processes depends on the specific signaling pathways that are activated; many of which include protein tyrosine kinases. These pathways include the mitogen-activated protein kinase pathway, Janus kinases/signal transducers and activators transcription pathway, spleen tyrosine kinase signaling, and the nuclear factor κ-light-chain-enhancer of activated B cells pathway. Many drugs are in development to target tyrosine kinases for the treatment of RA. Based on the number of recently published studies, this manuscript reviews the role of tyrosine kinases in the pathogenesis of RA and the potential role of kinase inhibitors as new therapeutic strategies of RA.

## Introduction

Rheumatoid arthritis (RA) is a destructive, inflammatory, polyarticular joint disease with an etiology that remains to be fully elucidated. RA is characterized by massive synovial proliferation and subintimal infiltration of inflammatory cells, followed by the destruction of cartilage and bone [1]. A number of cellular responses are involved in the pathogenesis of RA, including activation of inflammatory cells and expression of various cytokines. Macrophages, T cells, B cells and neutrophils migrate into synovial tissue and activate these cells to produce both inflammatory and degradative mediators that break down the extracellular matrix of cartilage. Synovial cells undergo hyperplasia, and angiogenesis occurs in synovial tissues further promoting inflammation. Although synoviocyte proliferation contributes to the hyperplastic response of synovial tissue in RA, synovial tissue hyperplasia must also be a result of the recruitment, retention and attachment of mast cells, neutrophils, and lymphocytes to the inflamed tissue as well as the stimulus of neoangiogenesis. Inflammatory mediators such as interleukin-6 (IL-6), interleukin-1 (IL-1) and tumor necrosis factor-α (TNF-α) play important roles in the pathogenesis of RA. These cytokines are abundant in synovial tissues and fluid from RA patients and overexpression of the cytokines mentioned above promote chronic inflammation and joint destruction [2]. These cytokines have emerged as dominant pro-inflammatory mediators and important molecular targets for therapy [3]. TNF-a and IL-1 has been reported to stimulate synovial cells to release VEGF which has important role in the angiogenesis observed in RA pathology [4,5]. Classically, immune responses are regulated by two subtypes of CD4 + T helper (Th) cells, designated Th1 and Th2. Th1 cells produce interferon- (IFN-γ) and interleukin-2 (IL-2), which are known to be important mediators of organ-specific autoimmune disorders. On the other hand, Th2 cells produce the cytokines IL-4, IL-5, IL-9, and IL-13, which are responsible for promoting the development of atopic allergy. RA has been reported to be a Th1 and not a Th2 associated disorder. At present, RA is thought to be a Th1 and/or Th17 mediated disorder. Th17 cells are relatively new subset of helper T cells and are characterized by expression of RORγt as a master regulator gene as well as secretion of IL-17A, IL-17F, IL-21 and IL-22 [6]. Human T-regulatory (Treg) cells were reported to differentiate to IL-17 producing cells [7]. Treg and Th17 cells appear to be associated as both of these cells are induced by TGF-beta and also express Th17-associated trascription factor RORγt. Th1 cells are also associated with Treg cells and Th17 cells, as the gene encoding for T-bet, the master regulator of Th1 differentiation, was found to be in an active state, according to histone methylationmarks, in both Th17 and Treg cells [8]. This observation indicated that Th17 and Treg cells remain to have the potential to upregulate the expression of T-bet and to differentiate towards Th1 cells.

The activation and function of these inflammatory cells depend on specific signaling pathways; many of which involve protein tyrosine kinases. Proteins from the synovial tissue of RA patients have been reported to be extensively phosphorylated by intracellular tyrosine kinases, supporting the importance of tyrosine kinases in the pathogenesis of RA [9]. These pathways include the mitogen-activated protein kinase (MAPK) pathway, the Janus kinases (JAK)/signal transducers and activators of transcription (STAT) pathway, spleen tyrosine kinase (Syk) signaling, and the nuclear factor κ-light-chain-enhancer of activated B cells (NF-κB) pathway

In fact, many drugs are in development to target tyrosine kinases for the treatment of RA. Based on the number of recently published studies, this review focuses on the role of tyrosine kinases in the pathogenesis of RA and potential role of kinase inhibitors as new therapeutic strategies for RA.

### Mitogen-Activated Protein Kinases

**Figure 1:**
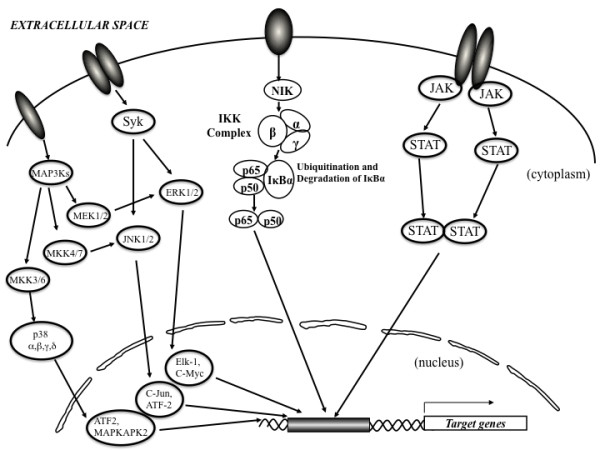
**Tyrosine kinases involved in the signal transduction pathways in rheumatoid arthritis**. Various tyrosine kinases have important roles in the pathogenesis of RA and these kinases are possible targets for the anti-RA strategy. MAP3K: MAPK kinase kinases, MKK: MAPK kinases, MAPK: mitogen-activated protein kinase, ERK: extracellular signal-regulated kinases, Syk: spleen tyrosine kinase, NF-κB: nuclear factor κ-light-chain-enhancer of activated B cells, IKK: IκB kinase, JAK: the Janus kinases, STAT: signal transducers and activators of transcription.

The serine/threonine MAPK pathway is activated in RA patients. Initiated by cytokine receptors, Toll-like receptors, and other danger signals, the pathway begins with the MAPK kinase kinases (MAP3K), which phosphorylate and activate the MAPK kinases (MKK), which in turn phosphorylate MAPK, leading to the activation of various transcription factors [10]. The MAPK include extracellular signal-regulated kinases (ERK) and the p38 kinase (p38). ERK1 and 2, activated by signaling from growth factor receptors and certain cytokine receptors, activate the transcription factors Elk-1 and c-Myc (Figure 1). The kinases p38α and p38β are typically activated by signaling from Toll-like receptors, as well as in response to oxidative stress, inflammatory cytokines, etc. The role of MAPK in transmitting signals from inflammatory cytokines such as TNF-α, which have proven to be successful targets in the treatment of RA, have made the MAPKs themselves attractive targets for the development of new therapies. However, the results from two 12-week studies on a p38α inhibitor, VX-702 (Vertex Pharmaceuticals; Cambridge, MA) were disappointing. Despite a trend toward an increased percentage of patients meeting the American College of Rheumatology (ACR) 20% improvement criteria (ACR20) in the treatment groups receiving the drug compared with placebo, the data were not statistically significant [11]. Furthermore, there was no discernable dose-dependent effect of the drug when patients were also treated with methotrexate. In addition, an increased incidence of adverse events was observed in the treatment (Cambridge, MA) groups compared with placebo, particularly skin rash. In addition, a slight increase in the percentage of patients with alanine transaminase (ALT) elevation was also noted. Pamapimod (RO4402257; Hoffmann-La Roche, Basel, Switzerland), a p38α inhibitor with very low p38β inhibitory activity, is another drug tested for potential efficacy against RA. In spite of the promising preclinical data with pamapimod, disappointing results in clinical studies are reported [12]. Thus far, inhibitors of MAP kinases have largely failed in clinical trials due to both lack of efficacy and adverse events. These results indicated that p38 may play a significant role in homeostasis as well as in disease states and its inhibition threrfore results in unacceptable side effects. Other targets in the MAPK pathway are also under investigation. ARRY-162 is an inhibitor of the MAPK extracellular signal-regulated kinase (MEK). Phase 1 studies demonstrated that this drug was able to inhibit 12-O-tetradecanoylphorbol-13-acetate-induced IL-1β TNF, and IL-6 production ex vivo. Phase 2 studies are under way in patients with RA. Some evidence suggests that in addition to inhibition of cytokine production, the drug blocks osteoclast differentiation and reduces bone resorption [13].

### Janus kinases

Janus kinase (JAKs) binds the cytoplasmic region of transmembrane-cytokine receptors. After receptor-ligand interactions, various JAKs are activated, resulting in tyrosine phosphorylation of the receptor and subsequent activation of STATs (signal transducer and activators of transcription), which act as transcription facotrs [14]. The STATs are phosphorylated by JAK, dissociate, dimerize via their SH2 domains, and translocate to the nucleus, where they initiate transcription of target genes (Figure 1). JAKs consist of four types: JAK1, JAK2, JAK3 and TyK2. Hormone-like cytokines (growth hormone, prolactin, erythropoietin (Epo), and thrombopoietin), GM-CSF, IL-3 and IL-5 use Jak2. IL-6, IL-10, IL-11, IL-19, IL-20 IL-22 and interferon (IFN)-γ use Jak1 and Jak2. Mice lacking Jak1 and Jak2 resulted in perinatal and embryonic lethality respectively. In contrast, humans and mice lacking Jak3 present with a distinct primary immunodeficiency disorder designated T-B+NK- severe combined immunodeficiency (SCID)[15,16]. Jak3 is the only Jak family member that associates with just one cytokine receptor-the common γ chain, which is exclusively used by the receptors for IL-2, IL-4, IL-7, IL-9, IL-15 and IL-21. These cytokines are critically involved in T and NK cell development and B cell function and proleferation [17]. By contrast, commonγchain-dependent cytokines are not important for the development or function of organs or tissue outside the immune system. Thus, a specific Jak3 inhibitor should have limited but precise effects on immune system cells and should leave other cell types unaffected. Tyk2 has broader functions than Jak3, as it binds to a number of cytokine receptor subunits. Tyk2-knockout mice are viable but have reduced sensitivity to interferons, IL-12 and IL-23. This results in increased susceptibility to some microbial pathogens. One patient with a Tyk2 deficiency has been described, whose cells were found to have impaired signaling in response to type I IFNs, IL-12, IL-23, IL-10 and IL-6. This patient had a primary immunodeficiency disorder characterized as a hyper-IgE-like syndrome with multiple, opportunistic infections with viruses, bacteria and fungi [18]. Suppressor of cytokine signaling (SOCS) proteins and cytokine inducible SH2-containing (CIS; also known as CISH) protein molecules comprise a family of intracellular proteins, several of which have been shown to regulate the responses of immune cells to cytokines.8-10 There are eight CIS/SOCS family proteins; CIS, SOCS1, SOCS2, SOCS3, SOCS4, SOCS5, SOCS6, and SOCS7. In addition to their ability to suppress signaling by ubiquitin-mediated degradation of the signaling complex, both SOCS1 and SOCS3 can inhibit JAK tyrosine kinase activity directly through their kinase inhibitory region (KIR), which has been proposed to function as a pseudosubstrate and which is important for the suppression of cytokine signals. Il-6 is one of the cytokines important for RA pathology and Il-6 binds to the IL-6 receptor α chain and to gp130. SOCS3 is a relatively specific inhibitor of gp130 and SOCS3 has been shown to negatively regulate Th17 development by the suppression of STAT3 activated by IL-6 or IL-23 [19]. There is a report showing that overexpression of SOCS3 by adenovirus gene transfer prevented the development of experimental arthritis [20]. Because of the significant role IL-6 plays in RA pathogenesis, and other evidence suggesting that the JAK/STAT pathway contributes to the disease, several JAK inhibitors have been developed and clinical trials are under way [21]. The most studied candidate in this pathway is CP- 690,550 (Pfizer; New York, NY), a small molecule that predominantly blocks JAK3 [22]. CP-690 550 is an orally available JAK antagonist and is a potent, selective inhibitor of the JAK family of kinases with selectivity for JAK1/3 over JAK2. Double blind randomized, placebo controlled clinical trials (RCTs) evaluating CP-690 550 have been conducted. In one study, 264 patients were randomized equally to receive placebo, 5 mg of CP-690,550, 15 mg of CP-690,550, or 30 mg of CP-690,550 twice daily for 6 weeks, and were followed up for an additional 6 weeks after treatment. By week 6, the ACR20 response rates were 70.5%, 81.2%, and 76.8% in the 5 mg, 15 mg, and 30 mg twice daily groups respectively, compared with 29.2% in the placebo group (P < 0.001). Improvements in disease activity in CP-690,550-treated patients compared with placebo were seen in all treatment groups as early as week 1. No opportunistic infections or deaths occurred [23]. In another study, patients were randomized equally to placebo, CP-690,550 5, 15 or 30 mg twice daily for 6 weeks, with 6 weeks' follow-up. The patient's assessment of arthritis pain (pain), patient's assessment of disease activity, Health Assessment Questionnaire-Disability Index (HAQ-DI) and Short Form-36 (SF-36) were recorded. At week 6, significantly more patients in the CP-690,550 5, 15 and 30 mg twice-daily groups experienced a 50% decrease in pain compared with placebo, clinically meaningful reductions in HAQ-DI and clinically meaningful improvements in SF-36 domains and physical and mental components [24]. Taken together, JAK inhibitors are promising for the treatment of RA and further clinical studies are ongoing.

### Spleen tyrosine kinase (Syk)

Syk is one of the important non-receptor-type protein tyrosine kinases (PTKs) and was originally isolated from a porcine spleen cDNA library. Syk is widely expressed in cells of the hematopoietic system;most notably in B cells. Various specialized domains such as Src homology 2 (SH2), SH3 and SH4 are defined by non- receptor PTKs and are involved in the regulation of PTK activity and interaction with other molecules in signaling pathway. Tandem SH2 domains of Syk family PTKs selectively bind to diphosphorylated immunoreceptor tyrosine-based activating motif (ITAM) of the cytoplasmic region of immune receptors, such as the T and B cell receptors. Cbl functions as a ubiquitin ligase towards Syk and that ubiquitylated Syk is targeted for proteasomal degradation, resulting in act as a negative regulator of Syk [25]. Because of its apparent critical role in regulating T-cell and B-cell expansion and the proliferation of cells containing the Fcγ-activating receptor as well as mediating immunoreceptor signaling in inflammatory cells and immune complex-mediated signal transduction, SyK must be considered a promising target for designing interventional drugs for the treatment of immune-mediated inflammatory arthritis. Syk activation activates downstream MAPKs and PI3K, resulting in the increase of IL-6 and MMP production (Figure 1). Syk is present in the synovium of patients with RA, and activation of Syk is important for cytokine and metalloproteinase production induced by TNF-α in fibroblast-like synoviocytes from patients with RA. In murine model of human RA, Pine et al. showed that R788(fostamatinib disodium), an oral prodrug that is rapidly converted to a potent and relatively selective inhibitor of Syk (R406) suppressed the severity of arthritis, bone erosions, pannus development and synovitis in murine collagen-induced arthritis (CIA)[26]. The reduced expression of SyK in the R788-treated mice correlated with an amelioration of clinical arthritis, a reduction in proinflammatory chemokines and cytokines, including the CXCR2 ligand KC-GRO-α, macrophage chemoattractant protein-1 (MCP-1), IL-1, and IL-6, as well as inducing suppression of cartilage oligomeric matrix protein release, the latter protein a sensitive in vitro biomarker for articular cartilage extracellular matrix degradation. Because of the apparent successful preclinical response to R788 in the well-validated CIA animal model of RA, a Phase II clinical trial involving, fostamatinib (R406), was conducted.. In addition to improving clinical outcomes measurements as determined by the American College of Rheumatology (ACR) criteria as early as 1 week after oral administration, R406 also reduced the level of serum IL-6 and MMP-3 (stromelysin-1) in the groups receiving 100 mg and 150 mg of R406 twice daily [27].

In a previous 12-week, ascending-dose, randomized, placebo-controlled Trial 4 (http://ClinicalTrials.gov number, NCT00326339) involving 189 patients who had active RA despite methotrexate therapy, a significant reduction in arthritis activity and in serum levels of interleukin-6 and matrix metalloproteinase 3 were seen in the two groups that received the highest doses of R788 (100 mg twice daily and 150 mg twice daily), as compared with the groups that received placebo or the 50-mg dose of R788 twice daily. Notable adverse events were diarrhea, neutropenia, and an elevation of blood pressure. In another study to assess the efficacy and safety of R788, 229 patients with active RA who had currently or previously failed a biologic therapy were enrolled in a 3-month double-blind, placebo-controlled trial of R788. In this study, the primary endpoint was the ACR 20 response at Month 3. Secondary endpoints included changes in inflammation and damage assessed by MRI, and changes in DAS. The ACR 20 response in the R788 100 mg twice daily (BID) group was 38% versus 37% in the placebo group at month 3. No significant differences were achieved in the ACR 20/50/70 levels at 3 months. There were differences between the groups in secondary endpoints from Baseline to month 3 in CRP and synovitis scores on MRI. There were baseline differences in steroid use, prior biologic use, and synovitis scores by MRI between the R788 group versus placebo which may have affected the outcomes. A high placebo response rate was seen in this trial and exploratory analysis suggests this may in part have been driven by patients who entered the trial with an elevated erythrocyte sedimentation rate, but normal c-reactive protein [28]. A phase 2 study was conducted to evaluate the efficacy and safety of R788, in patients with active RA despite methotrexate therapy. Patients (458) who had active RA despite long-term methotrexate therapy in a 6-month trial were enrolled in a double-blind, placebo-controlled trial. The primary outcome was the ACR20 response at month 6. A clinically significant effect was noted by the end of the first week of treatment. Adverse effects included diarrhea, upper respiratory infections, hypertension and neutropenia. This study indicated that R788 reduced disease activity in patients with RA with adverse events including diarrhea, hypertension, and neutropenia [29]. Although R788 was effective in the reduction of disease activity in patients with RA, there is a high potential for adverse events including neutropenia and infections. As Syk has been reported to have a role in hematopiesis, neutropenia side effects can be explained by the inhibition of Syk [30]. In addition, the specificity of the kinase inhibitor is doubtful as the active form of this kinase inhibitor (R406) has been reported to also target the Lyn and Lck kinases in addition to Syk [31]. As Lck is a critical kinase in T cell lineage commitment, inhibition of Lck might suppress immunity, resulting in a high risk for infections [32]. Furthermore, as suppression of Lyn has been reported to play a role in an antibody-mediated autoimmune disease resembling systemic lupus erythematosus in mice, inhibition of Lyn might increase the risk for the development of lupus [33]. On the other hand, R788 has been shown to reduce the development of nephritis in model mice of human lupus [34]. Therefore, as we indicated, further studies are indeed needed to assess the safety of this Syk inhibitor [35].

### Kinases involved in NF-κB signaling pathway

The NF-κB family of transcriptional activators regulates the expression of a variety of cytokines involved in the pathology of RA, including IL-1, TNF-α, and IL-6 [36]. In most cells, NF-κB complexes are located largely in the cytoplasm. In response to pro-inflammatory cytokines such as TNF-α and IL-1β, the inhibitory proteins IκB, become phosphorylated by the IκB kinase (IKK) complex on two serine residues located in the N-terminal region [37], which results in their rapid ubiquitination and proteolysis by the 26S proteasome, thereby allowing the liberated NF-κB to translocate to the nucleus (Figure 1)[38]. The IKK complex consists of two catalytic subunits, IKKα and IKKβ, and a regulatory subunit IKKχ [39-42]. The kinase activity of both IKKα and IKKβ is induced by a wide variety of NF-κB inducers such as TNF-α or IL-1β, and mediated by upstream kinases including NIK and the extracellular signal-regulated kinase kinase kinase 1, 3 [43-45].

The role of NF-κB in the pathogenesis of RA has been described previously. Mice lacking functional NF-κB-inducing kinase have been shown to be resistant to antigen-induced arthritis [46]. Constitutively active STAT-6, which blocks NF-κB activation has also been shown to inhibit inflammatory arthritis in mice [46]. Local treatment with the selective IκB kinase beta inhibitor NEMO-binding domain peptide has been shown to ameliorate rat adjuvant arthritis [47]. We also have shown that a peroxisome proliferator-activated receptor-alpha ligand, fenofibrate, inhibited rat adjuvant arthritis [48]. Therefore, NF-κB, which is responsible for the production of inflammatory molecules, as well as for the differentiation of osteoclasts, is an important target for RA therapy. Fasudil hydrochloride (Fasudil) (1-(5-Isoquinolinesulfonyl)-homopiperazine hydrochloride) is a serine/threonine kinase inhibitor [49,50] and is the first kinase inhibitor drug used in a clinical setting in Japan [51]. Fasudil has been used for years for the treatment of subarachnoid hemorrhage and its safety in clinical settings is well established. Fasudil has been reported to inhibit NF-κB signaling following infection by the human immunodeficiency virus [52].

We found that systemic administration of fasudil, a novel serine/threonine kinase inhibitor, inhibits the development of adjuvant-induced arthritis in rats through the inhibition of the NF-κB activation pathway. We demonstrated that fasudil inhibites cytokine production from fibroblast-like synoviocytes, adhesion molecule expression on human endothelial cells in culture, and inhibition of NF-κB without showing inhibition of either IκBα degradation or nuclear translocation of NF-κB. However fasudil inhibited IL-1β induced NF-κB transactivation as well as DNA binding of NF-κB. In addition, in vivo studies have shown that fasudil suppresses the development of arthritis in an adjuvant-induced arthritis model [53]. These results indicate that fasudil inhibits the NF-κB signaling required for the binding of NF-κB to specific DNA sequences. Consequently our results indicate that fasudil might function by inhibiting the phosphorylation of p65 or a novel NF-κB kinase [53].

## Conclusion

The lack of clinical efficacy and the high rate of adverse events seen in the p38 MAPK inhibitor trials highlight several problems in designing drugs that target these critical intracellular signaling pathways. First, the structural similarity of many kinases calls into question the true specificity of the drugs that target them. Off-target effects may account for many of the adverse effects observed. Second, the importance of these pathways in host defense against disease has naturally resulted in signaling redundancy. Thus, inhibition of one signaling component may be compensated for by the modification of complementary pathways. Therefore, lack of specificity may result in off-target effects causing increased side effects, however absolute specificity may result in a lack of efficacy due to redundancy in signaling. Towards increasing kinase inhibitor specificity, we previously reported that a pseudo-substrate peptide for cyclin-dependent kinase 7 inhibits transcriptional activation by the Tat protein of the human immunodeficiency virus [54,55]. A similar approach may be useful in the inhibition of kinases important for RA therapy.

Cellular signal transduction pathways including various transcription factors play critical roles in regulating the functions of immune effector cells, including expression of cytokines/chemokines and also in the control of synovial cell apoptosis. Growing experimental evidence emphasizes the importance of NF-κB, NFAT, JAK/STAT and other transcription factors in RA. Therefore, signaling cascades associated with these transcription factors are possible targets for a comprehensive anti-RA strategy. New therapeutic strategies may target transcription factor activity by controlling their synthesis or modulating protein-protein interactions in the activating signaling cascade. Specific inhibitors have already reported, for example: a small molecule inhibitor of NFAT, decoy oligonucleotides for NF-κB, interfering RNAs targeting components of the STAT pathway, and inhibition of Toll-like receptor signalling pathway by Chaperonin 10 [56-60]. However, most of the intracellular kinases that activate transcription factors involved in RA have pleiotropic roles in other biological processes and therefore, inhibition of these transcription factors might invite unexpected side effects *in vivo*. Clinical and molecular studies must be undertaken in tandem in order to develop effective and safe therapeutic strategies against RA. It is our hope that an orally administered, small molecule tyrosine kinase inhibitor, with minimal adverse side-effects will be available in the near future for treatment of RA. This treatment should be cheaper than existing biologic-based treatments and improve disease outcomes leading to greater patient acceptance.

## Competing interests

The authors declare that they have no competing interests.

## Authors' contributions

HO and AK wrote the manuscript and approved the final manuscript.
